# Supplementary figures and images for: pLM-BLAST: distant homology detection based on direct comparison of sequence representations from protein language models
Source: Bioinformatics. 2023 Sep 19;39(10):btad579. doi: 10.1093/bioinformatics/btad579 (PMC10576641; doi:10.1093/bioinformatics/btad579)

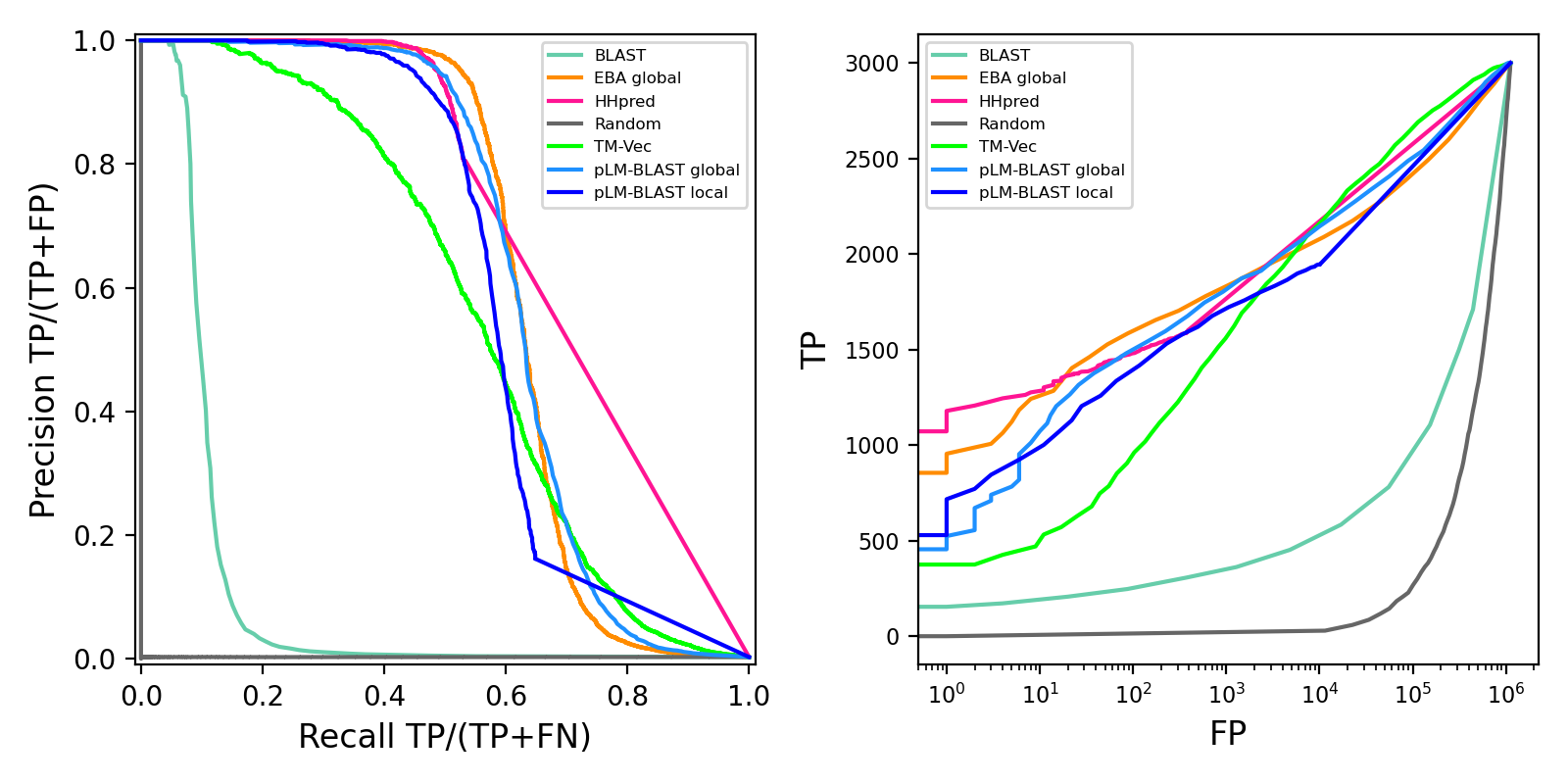

Supplement: btad579_Supplementary_Data [file btad579_supplementary_data.zip › Supplementary_Figure_1.png]
